# Supplementary material for: Sonographic Anatomy and Normal Measurements of the Human Kidneys: A Comprehensive Review
Source: Diagnostics (Basel). 2025 Dec 15;15(24):3208. doi: 10.3390/diagnostics15243208 (PMC12731967; doi:10.3390/diagnostics15243208)
Supplement: Supplementary file 1 [file diagnostics-15-03208-s001.zip › SS2.pdf]

Diagnostic algorithm for renal cystic masses

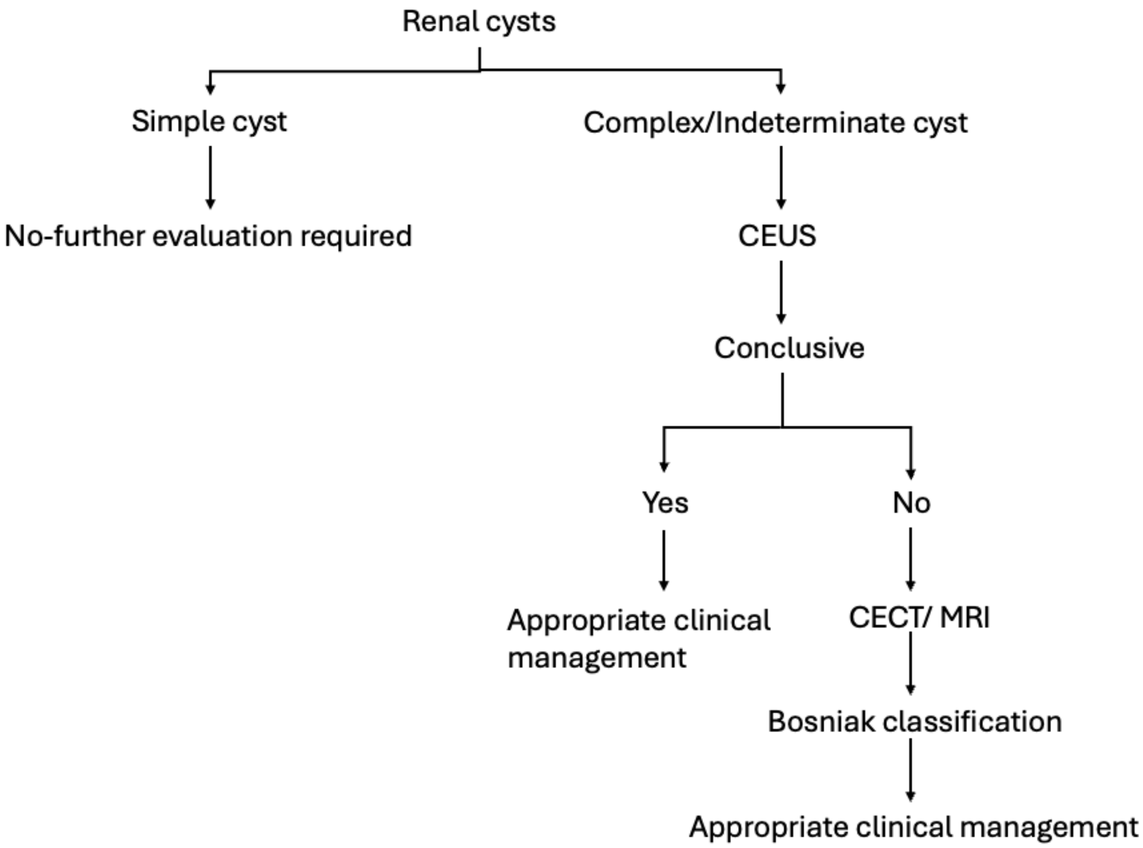

CEUS: Contrast-enhanced ultrasound

CECT: Contrast-enhanced computed tomography

MRI: Magnetic resonance imaging
