# Supplementary material for: Sonographic Anatomy and Normal Measurements of the Human Kidneys: A Comprehensive Review
Source: Diagnostics (Basel). 2025 Dec 15;15(24):3208. doi: 10.3390/diagnostics15243208 (PMC12731967; doi:10.3390/diagnostics15243208)
Supplement: Supplementary file 1 [file diagnostics-15-03208-s001.zip › SS1.pdf]

Boolean Search String

Serial Number	Database	Search String	Notes
1.	NCBI PubMed	(("kidney"[MeSH Terms] OR "renal"[All Fields]) AND ("ultrasonography"[MeSH Terms] OR "ultrasound"[All Fields] OR "sonography"[All Fields]) AND ("anatomy"[All Fields] OR "normal"[All Fields] OR "reference values"[MeSH Terms] OR "echogenicity"[All Fields] OR "cortical thickness"[All Fields] OR "parenchymal thickness"[All Fields] OR "Doppler"[All Fields] OR "resistive index"[All Fields])) NOT (animals[MeSH Terms] NOT humans[MeSH Terms])	• Search was conducted between March 2024-May 2025.<br>• Filters: Humans, English, 1950-Present<br>• Exploded MeSH terms were allowed.
2.	Embase, Elsevier	('kidney'/exp OR renal.) AND ('ultrasonography'/exp OR ultrasound. OR sonography) AND (normal OR anatom. OR 'reference value'/exp OR echogenicity OR 'renal cortex thickness'. OR 'parenchymal thickness' OR 'resistive index') NOT ('animal'/exp NOT 'human'/exp)	
3.	Scopus, Elsevier Scopus	(TITLE-ABS-KEY (kidney OR renal) AND TITLE-ABS-KEY (ultrasound OR ultrasonography OR sonography) AND TITLE-ABS-KEY (normal OR anatomy OR	• Search was limited to only key words and title fields

		"reference values" OR echogenicity OR "cortical thickness" OR "parenchymal thickness" OR "resistive index" OR Doppler)) AND (LIMIT-TO (LANGUAGE, "English"))	
4.	Web of Science Core Collection, Clarivate	TS = ((kidney OR renal) AND (ultrasound OR ultrasonography OR sonography) AND (normal OR anatomy OR "reference values" OR echogenicity OR Doppler OR "cortical thickness" OR "parenchymal thickness" OR "resistive index")) Refined by: Document Type = Article Language = English	• Topic search included: Title, Abstract, keywords, and keywords plus

Please note: All search strings were copied, and relevant data were recorded in EndNote and Zotero reference management tools.
